# Protocol: Factors contributing to the discontinuation of breastfeeding upon women's return to work: A systematic review protocol

**DOI:** 10.1002/cl2.1434

**Published:** 2024-09-09

**Authors:** Ana Veronica Scotta, Paula Eugenia Barral, Ailin Farre, Elio Andrés Soria

**Affiliations:** ^1^ Instituto de Investigaciones en Ciencias de la Salud (INICSA), Consejo Nacional de Investigaciones Científicas y Técnicas Córdoba Argentina; ^2^ Escuela de Fonoaudiología, Facultad de Ciencias Médicas Universidad Nacional de Córdoba Córdoba Argentina; ^3^ Cátedra de Biología Celular, Histología y Embriología, Facultad de Ciencias Médicas Universidad Nacional de Córdoba Córdoba Argentina; ^4^ Instituto de Biología Celular, Facultad de Ciencias Médicas Universidad Nacional de Córdoba Córdoba Argentina

**Keywords:** breast feeding, postpartum period, return to work, systematic review, women, working

## Abstract

This is the protocol for a Campbell systematic review. The objectives are as follows. In order to understand the variables affecting breastfeeding in working women, this systematic review will aim to determine the factors associated with early breastfeeding cessation upon women's return to work within a Social‐Ecological framework. This will be achieved by answering the following questions: Which individual factors are associated with early discontinuation of breastfeeding upon returning to work?; Which interpersonal factors are associated with early discontinuation of breastfeeding upon returning to work?; Which community factors are associated with early discontinuation of breastfeeding upon returning to work?; Which institutional factors are associated with early discontinuation of breastfeeding upon returning to work?; Which public policies are associated with early discontinuation of breastfeeding upon returning to work?

## 1 BACKGROUND

Breastfeeding provides optimal nutrition for infants, producing a myriad of benefits reaching beyond infancy to improve child and adult health. In this sense, breastfeeding improves infant survival, lowers the incidence and severity of infectious diseases (such as gastrointestinal and respiratory infections), improves cognitive outcomes, and lowers the rates of chronic illnesses such as obesity and type II diabetes (North et al., 2022). In this sense, supporting breastfeeding initiation and maintenance constitutes an effective health policy, especially for the low and middle‐income countries that suffer most of the societal and economic costs of infant morbimortality (Quesada et al., 2020). Consequently, the World Health Organization (WHO) recommends exclusive breastfeeding during the first 6 months of life, and continued breastfeeding for at least 2 years of age.

Despite the numerous recognized health, societal, economic, and environmental advantages associated with breastfeeding, women encounter multiple barriers that impede this practice, resulting in suboptimal breastfeeding coverage across the world (Patil et al., 2020; Tomori, 2022). The determinants influencing breastfeeding choices are inherently intricate and multifaceted. Consequently, a holistic framework is necessary to understand the complexities faced by these women upon their return to work (Dunn et al., 2015). To address this, the Social‐Ecological Model provides a comprehensive conceptual framework to assess the relationships between people and their environment. This model postulates that health behaviors are shaped by the dynamic interplay of various environmental domains, encompassing individual, interpersonal, community, institutional, and policy‐making spheres of influence (Snyder et al., 2021).

Breastfeeding has proven to be a protective factor for various diseases in women, such as anemia, breast and ovarian cancers, type II diabetes, hypertension, cardiovascular diseases, and mental illnesses (Minchala‐Urgiles et al., 2020; Rivi et al., 2020). Consequently, promoting the holistic health of caregiving women involves promoting human lactation. This could contribute to reducing the gap in the level of self‐perceived quality of life between men and women (Gumà et al., 2015; Larrañaga et al., 2008). A woman's decision to breastfeed depends on multidirectional factors operating at every level of the Social‐Ecological Model. Notably, breastfeeding, akin to most health outcomes, is profoundly impacted by social and structural determinants that may not be apparent to lactating families (Tomori, 2022). Existing research has identified barriers to breastfeeding at each one of these levels, including prior experience, mental health status, time constraints, low self‐efficacy, education, and occupational characteristics at the individual level. At the interpersonal level, challenges manifest as insufficient support from family, friends, and colleagues. The community level includes issues such as the lack of normalization of breastfeeding practices in public, and limited access to community resources. Institutional barriers involve insufficient hospital resources, inappropriate practices concerning infant formula provision, and inadequate workplace accommodations, among others. Policy‐level challenges comprise scarce workplace protections (e.g., parental leave) and breastfeeding‐promoting legislation. Additionally, issues such as the gender pay gap and higher rates of informal employment in women compared to men have their origin in multiple levels of influence (Dunn et al., 2015; Snyder et al., 2021; Vilar‐Compte et al., 2021).

In particular, the workplace emerges as a complex environment where breastfeeding practices are greatly influenced by the interplay of multiple factors at various levels. Specifically, organizational support at the institutional level and interpersonal support from colleagues and supervisors have been identified as critical factors that influence individual‐level factors in working women, such as their intention to breastfeed and perceived self‐efficacy (Vilar‐Compte et al., 2021). This underscores the pivotal role of supportive workplace environments in protecting breastfeeding among employed women, taking into account their particular needs regarding their job position and working conditions. Regarding this, thousands of women work independently or under informal conditions worldwide, which increases their risk of discontinuing breastfeeding. Thus, they face special challenges that need to be addressed across all spheres of influence (Chowdhury et al., 2021; Landour, 2020; Ulep et al., 2021).

Studying the factors that hinder women from breastfeeding upon their return to work is crucial for several strong reasons. Firstly, despite the existence of numerous national and international policies addressing the issues that enforce women's right to breastfeed, their practical implementation remains heterogeneous and insufficient (Hauck et al., 2021; Vilar‐Compte et al., 2022). In this sense, policies such as paid maternity leave and breaks during the workday are widely implemented across the world, but their design might be inadequate for less‐studied groups of women, who are more vulnerable due to a lower socioeconomic status, informal employment, or gender discrimination (Pérez‐Escamilla, 2020; Vilar‐Compte et al., 2022). Consequently, there is a need to redirect these policies, making them more assertive and responsive to the challenges faced by all working women.

Given this, generating comprehensive studies on the barriers to breastfeeding is of critical significance to avoid generic or fragmented policy approaches, leading to better‐targeted interventions with a higher likelihood of success. In this sense, achieving the Sustainable Development Goals (SDGs) in the medium term requires these strategies to be more efficient. Addressing obstacles to breastfeeding is integral to the broader goal of promoting maternal and child health, contributing to several SDGs, including those related to “Good Health and Well‐being” and “Gender Equality” (De Souza et al., 2021). Finally, supporting women in maintaining breastfeeding can enhance female workforce productivity and contribute to a healthier and more sustainable economy, leading to long‐term economic growth (De Souza et al., 2023).

Some evidence‐synthesis studies previously examined the topic of breastfeeding in women returning to work. Dutheil et al. (2021) conducted a systematic review and meta‐analysis on the prevalence of breastfeeding after returning to work, finding an association between a country's economic status and the prevalence of exclusive, but not partial, breastfeeding. Nonetheless, the authors found limited information about key factors such as sociodemographic variables, workplace support, and relevant policies, which at the time of publishing impeded the meta‐analysis of their impact on breastfeeding practices. Nurjanah et al. (2023) aimed to identify predictors of breastfeeding in mothers who returned to work through a systematic review. They reported a correlation between part‐time employment and delayed return to work with extended breastfeeding duration. Nevertheless, the study shows notable methodological limitations, including the absence of clearly defined inclusion and exclusion criteria, failure to report the search strategy, and a lack of a risk‐of‐bias assessment, resulting in the potential exclusion of pertinent evidence and the generation of biased conclusions. On the other hand, Chang et al. (2021) performed a systematic review of qualitative evidence on working women's and employer's experiences. In this sense, protective factors included personal motivation, interpersonal and institutional support, access to childcare services, and relevant legislation. Trego et al. (2021) performed a scoping review following the Social‐Ecological model limited to active duty military women in the United States, whereas De Souza et al. (2023) focused on organizational and policy‐level conditioners to breastfeeding and their impact on the fulfillment of the SDGs. Finally, the reviews performed by Litwan et al. (2021), Nardi et al. (2020), Dinour and Szaro (2017), and Abdulwadud and Snow (2012) focused exclusively on the institutional sphere reporting workplace interventions that support breastfeeding. To the authors' best knowledge, no systematic review, meta‐analysis, or review protocol of quantitative evidence exists that covers factors associated with breastfeeding cessation upon women's return to work within a Social‐Ecological framework.

The development of a protocol for systematic revision is a determinant in accomplishing several criteria of information quality, such as rigor and transparency. The standardization process provides a detailed and structured guide for conducting evidence synthesis by clearly defining the objectives, inclusion and exclusion criteria, data sources, search strategies, and analysis methods. This ensures that the review is conducted systematically and consistently, which can be followed by other researchers to collaborate, provide feedback, evaluate, or replicate the study and verify the outcomes, thereby increasing its credibility and reliability. Furthermore, it minimizes the risk of selection and publication biases, as all studies are evaluated using the same criteria, ensuring that the search strategy is comprehensive and no relevant studies are omitted. Additionally, it optimizes time and resource management, teamwork, and progress tracking. Thus, by following the protocol, a systematic review can identify and synthesize the best available evidence to generate more robust conclusions based on solid data, which is crucial for informing health policies and practices that support breastfeeding by considering its benefits and determinants to propose key protective interventions (Kugley et al., 2016; Tufanaru et al., 2020).

## 2 OBJECTIVES

In order to understand the variables affecting breastfeeding in working women, this systematic review will aim to determine the factors associated with early breastfeeding cessation upon women's return to work within a Social‐Ecological framework. This will be achieved by answering the following questions:

- Which individual factors are associated with early discontinuation of breastfeeding upon returning to work?
- Which interpersonal factors are associated with early discontinuation of breastfeeding upon returning to work?
- Which community factors are associated with early discontinuation of breastfeeding upon returning to work?
- Which institutional factors are associated with early discontinuation of breastfeeding upon returning to work?
- Which public policies are associated with early discontinuation of breastfeeding upon returning to work?

## 3 METHODS

This protocol follows the Methodological Expectations of Campbell Collaboration Intervention Reviews (MECCIR) conduct standards, as suggested by The Methods Group of the Campbell Collaboration (2017) (Supporting Information S1: I). The proposed review will be conducted following the JBI methodology for systematic reviews of etiology and risk, which aims to identify and synthesize the available evidence on the factors that are associated with a health outcome (Moola et al., 2020), and the NIRO‐SR guidelines for the development of non‐intervention, reproducible, and open systematic reviews (Topor et al., 2022). A timetable for the review process is presented in Supporting Information S2: II.

### 3.1 Criteria for considering studies for this review

#### 3.1.1 Types of studies

Articles based on quantitative methods will be included in the review to answer all review questions. Gray literature sources, such as conference proceedings, theses, dissertations, and other texts will also be considered for inclusion in this review to develop a comprehensive overview of all available evidence. Study designs include randomized or nonrandomized controlled trials, pre‐post studies, longitudinal studies (case‐control or cohort studies), and analytical cross‐sectional studies. Evidence syntheses (such as systematic reviews) will be excluded to avoid data duplication, but their reference lists will be screened for primary sources of interest.

#### 3.1.2 Types of participants

This review will consider studies that include cisgender women who breastfeed and return to work. No restrictions regarding age will be applied. Any form of paid work performed by breastfeeding women will be considered. This includes formal or informal, dependent or self‐employed, and full or part‐time work. Data focusing on unpaid care and domestic work activities will not be included in the analysis.

#### 3.1.3 Types of exposure

This review will consider studies about all potential individual, interpersonal, community, institutional, and public‐policy factors associated with the outcome of interest. Given the holistic and multifaceted nature of the Social‐Ecological Model, overlaps among these spheres are anticipated. In order to manage these overlaps, we will employ an approach that involves the identification of the primary level of influence for each factor, the context in which each factor exerts its influence, and the supporting literature. Additionally, we will clearly report and discuss these intersections (Caperon et al., 2022). Thus, the use of this theoretical framework will provide a multi‐level lens to reflect the complex interplay among the factors that affect the decision to breastfeed, taking into account its previous application in research about breastfeeding barriers and facilitators (Dunn et al., 2015; Ma et al., 2018; Snyder et al., 2021).

#### 3.1.4 Types of outcome measures

This review will consider studies that explore early breastfeeding cessation. Any form of breastfeeding (exclusive or partial) will be considered. Exclusive breastfeeding involves feeding infants only human milk, with no additional liquid or solid foods. Partial breastfeeding, conversely, refers to the infant feeding practice that combines human milk and other forms of nutrition, such as infant formula.

The WHO recommends exclusive breastfeeding during the first 6 months of life. After that, complementary foods are gradually introduced while breastfeeding continues up to 2 years of age or beyond. Thus, early cessation will be defined as discontinuing breastfeeding against the WHO recommendations. Studies that do not meet this criterion will be excluded from the review.

##### Primary outcomes

Early breastfeeding cessation associated with labor, defined as follows:

- For infants younger than 6 months who are exclusively breastfed: cessation of exclusive breastfeeding (including transition to partial breastfeeding).
- For infants younger than 6 months who are not exclusively breastfed: cessation of partial breastfeeding.
- For infants aged between 6 months and 2 years: cessation of partial breastfeeding.

##### Secondary outcomes

None.

#### 3.1.5 Duration of follow‐up

Given the broad scope of this review, no time restrictions on breastfeeding cessation will be pre‐established. If sufficient evidence is found, the timing of the return to work will be categorized for subgroup analysis as follows: <1 month, 1–3 months, >3–<6 months, and ≥6 months after birth.

#### 3.1.6 Types of settings

No further socio‐geographical limitations will be defined.

### 3.2 Search methods for identification of studies

The guide to information retrieval for Campbell systematic reviews was consulted to organize the search for studies (Kugley et al., 2016). Within this framework, the search strategy was designed to identify studies published before May 2024, and the keywords selected will be agreed upon by all authors, following a three‐step methodology (Moola et al., 2020). A professional librarian was consulted during all stages of the search strategy design. First, three databases (PubMed, The Cochrane Library, and Epistemonikos) were searched for key terms used in the title, abstract, and indexation terms. Secondly, a full search strategy was developed for PubMed (MEDLINE) including all selected keywords and indexation terms (Supporting Information S3: III). Criteria of search sensitivity maximization, precision, and manageability were considered during the development of this strategy (Paez, 2017). The resulting search strategy will then be adapted for all other information sources. Finally, a manual search will be conducted in the reference lists of identified reviews for additional evidence sources. Articles must be written in English or Spanish to be included in the analysis. If the revision is performed by foreign language authors, the content consistency of these articles should be addressed by a scientific translator acknowledged in the text. No date restrictions will be applied.

#### 3.2.1 Electronic searches

The following databases will be used for the search:

- MEDLINE: accessed through PubMed and Ovid
- SciELO
- Scopus
- APA PsycInfo
- Cumulative Index of Nursing and Allied Literature (CINAHL)
- Embase
- The Campbell Collaboration Library
- The Cochrane Library
- Epistemonikos
- Latin American and Caribbean Health Sciences Literature (LILACS): accessed through Global Index Medicus
- Index Medicus for the Eastern Mediterranean Region (IMEMR): accessed through Global Index Medicus
- Index Medicus for South‐East Asia Region (IMSEAR): accessed through Global Index Medicus
- Western Pacific Region Index Medicus (WPRIM): accessed through Global Index Medicus
- Abridged Index Medicus or “Core Clinical” (AIM): accessed through Global Index Medicus
- Academic Search Complete (accessed through EBSCOhost)
- Applied Science & Technology Full Text (H.W. Wilson) (accessed through EBSCOhost)
- Business Source Premier (accessed through EBSCOhost)
- Dentistry & Oral Sciences Source (accessed through EBSCOhost)
- Fuente Académica Premier (accessed through EBSCOhost)
- MedicLatina (accessed through EBSCOhost)
- Business Source Premier (accessed through EBSCOhost)

To complement the database searches, we will conduct hand searches of the following relevant journals: Journal of Human Lactation, Breastfeeding Medicine, and International Breastfeeding Journal. Additionally, experts in the field will be identified by selecting the corresponding authors of the articles included in the review. These experts will be consulted to provide any additional unpublished data, which will be incorporated into the analysis.

#### 3.2.2 Searching other resources

Gray literature sources include documents such as conference proceedings, dissertations, trial registers, and government reports (Kugley et al., 2016; Paez, 2017). Certain gray literature sources are included in the abovementioned databases and thus will be identified during the general search. Moreover, separate searches for gray literature sources will be conducted on the following databases:

- Trial registers will be searched on the Cochrane Controlled Register of Trials (CENTRAL), ClinicalTrials.gov, The WHO International Clinical Trials Registry Platform (ICTRP), and the EU Clinical Trials Register.
- Dissertations will be searched on EBSCO Open Dissertations and Open Access Theses and Dissertations (OATD).
- Documents from health organizations and government institutions will be identified using web searches on Google.com (using advanced search tools) and OpenMD.com, with the search restricted to.org and.gov domains (site:.org OR site:.gov).
- Conference proceedings will be identified in CINAHL, MEDLINE, Scopus, and PsycINFO, and complemented with hand searches performed on the following journals: BMC Proceedings, MDPI Proceedings, and The Open Conference Proceedings Journal (discontinued in 2020).
- Additionally, ResearchGate will be used for all gray literature documents.

### 3.3 Data collection and analysis

#### 3.3.1 Description of methods used in primary research

This review is expected to encompass primarily observational and analytical studies, given that these study designs are most frequently employed to assess the relationship between certain exposure variables and health outcomes (Moola et al., 2020). Additionally, some experimental and quasi‐experimental studies are expected to be included.

#### 3.3.2 Selection of studies

The study selection will be performed using the Rayyan software (Ouzzani et al., 2016), to collate and upload all identified records for duplicate detection and removal. The screening and selection process will be performed independently by two randomly assigned subjects from a three‐reviewer pool (AVS, AF, and PEB). The third reviewer will solve any disagreements that arise during the selection process. Firstly, titles and abstracts will be reviewed to determine whether they meet the inclusion criteria. Then, all potentially relevant sources will be retrieved in full. Reviewers will contact the corresponding author of all manuscripts of interest whose full text is unavailable, with a maximum of two reminders. Finally, inclusion criteria will be confirmed in the full texts. The reasons for the exclusion of studies will be recorded and reported in the systematic review manuscript following the PRISMA flowchart and guidelines (Page et al., 2021).

#### 3.3.3 Data extraction and management

Data extraction from studies that meet the inclusion criteria and their quality assessment will be independently performed by two reviewers using a tool developed ad hoc (Supporting Information S4: IV), encompassing title, authors, gender of authors, year of publication, risk of bias, population, exposure, outcome, study methods, and key findings relevant to the review questions. To ensure comprehensive data extraction, the developed tool will undergo pilot testing on a representative sample of articles (Moola et al., 2020), and it will be revised as necessary during the process. All modifications will be detailed in the final manuscript of the systematic review. Corresponding authors will be contacted to request missing or additional data when required, with a maximum of two reminders.

#### 3.3.4 Assessment of risk of bias in included studies

The methodological quality and risk of bias of randomized controlled trials and nonrandomized intervention studies will be evaluated with the Cochrane RoB 2 and ROBINS‐I tools, respectively (Sterne et al., 2016; Sterne et al., 2019). These tools focus on multiple domains, which are assessed through signaling questions that indicate the risk of different types of bias. Based on the answers to these questions, RoB 2 classifies studies as having low, high, or “some concern of” risk of bias (Sterne et al., 2019). ROBINS‐I follows a similar algorithm to classify the risk of bias as low, moderate, serious, and critical (Sterne et al., 2016).

Studies with observational designs will be assessed using the JBI Critical Appraisal Tools for cohort studies, case‐control studies, and analytical cross‐sectional studies, as appropriate (Moola et al., 2020). These instruments comprise checklists of questions stipulated by each tool, comprising four domains: internal validity, external validity, statistical conclusion validity, and comprehensiveness of reporting. Questions are answered with a response of “yes,” “no,” “unclear,” or “not applicable” (Barker et al., 2023). The risk of bias for each study will be interpreted as low (all items are rated as “yes”), unclear (at least one item rated as “unclear”), or high (at least one item rated as “no”) (Maguire et al., 2024).

Evaluations will be performed independently by two randomly assigned subjects from a three‐reviewer pool (AVS, AF, and PEB). The third reviewer will solve any disagreements that arise during the assessment process.

#### 3.3.5 Measures of treatment effect

The outcome of interest is early breastfeeding cessation, a dichotomous variable. Consequently, odds ratios and their 95% confidence intervals will be reported concerning the individual, interpersonal, community, institutional, and public‐policy variables under study. For numerical predictors, Cohen's *d* will be calculated and then transformed into an odds ratio.

Unlike risk ratios, odds ratios can be computed for any study design, making them optimal for assessing effect size for dichotomous data (Tufanaru et al., 2020). Given the potential for interpretational challenges, particular attention will be given to the reporting of odds ratios in the review manuscript to prevent confusion with risk ratios and ensure accurate interpretation by readers and stakeholders.

#### 3.3.6 Unit of analysis issues

In cluster studies, a unit‐of‐analysis error occurs when the unit of allocation differs from the unit of analysis, and data from individuals are analyzed as if no clustering had occurred. This error might lead to artificial small p‐values and false‐positive conclusions (Higgins, Li, et al., 2023). Study designs that can result in unit‐of‐analysis errors and are potentially eligible for this review include cluster randomized trials (such as those in which different workplaces or community settings are randomized to receive different interventions), cluster non‐randomized studies, individually randomized group treatment trials (where women are individually randomized but interventions are performed at the group level), multi‐arm studies (in which multiple intervention groups are assessed against a single control group), studies in which women undergo more than one intervention, and studies that measure the breastfeeding cessation at different time points (Higgins, Eldridge, et al., 2023; Thuesen et al., 2020).

In all cases, methods to account for clustering effects will be screened in the primary study design (e.g., randomly partitioning the control group for each intervention in multi‐arm studies). If potential unit‐of‐analysis issues were not addressed in each primary study, the consequent approach will involve aggregating data at the study level by combining multiple observations, clusters, or units into a single effect estimate (López‐López et al., 2018; Rücker et al., 2017). An alternative approach will be applying a three‐level model (Van den Noortgate et al., 2015), with robust variance estimation methods (Fernández‐Castilla et al., 2021; Tipton, 2015), in cases where multiple measurements of breastfeeding cessation are present within the same study (e.g., different time points or among different subgroups of women). If studies lack sufficient information for analysis, reviewers will contact the corresponding author for additional information with a maximum of two reminders. Finally, if no response is received or data is insufficient, studies will be excluded from the meta‐analysis and reported in the narrative synthesis.

#### 3.3.7 Criteria for determination of independent findings

As explained above, the reporting of multiple effect sizes on primary studies can introduce statistical dependency on meta‐analyses. Consequently, the following methods are proposed to address this issue:

- Studies with multiple outcomes: Only data regarding the outcome of interest (breastfeeding cessation) will be included in the meta‐analysis.
- Different data sources estimating effect sizes for the same sample of women: A three‐step process will be implemented: (1) if multiple study designs were performed on the same sample of women, the study with the highest level of evidence will be selected (e.g., a randomized controlled trial will be preferred over an observational study); (2) if multiple studies with similar designs exist, the study with the lowest risk of bias will be selected; (3) if multiple studies have a similar risk of bias, the most recent study will be included in the analysis.

#### 3.3.8 Dealing with missing data

If the selected studies do not provide the data necessary to compute odds ratios and 95% confidence intervals, or if any other relevant information is missing to fill the data extraction tool, reviewers will contact the corresponding author of the study with a maximum of two reminders. If this process is unsuccessful in obtaining sufficient data to include the study in the meta‐analysis, it will only be included in the narrative synthesis.

#### 3.3.9 Assessment of heterogeneity

Forest plots for the visualization of individual and pooled effect sizes on breastfeeding cessation will be presented and accompanied by the *Q* test of homogeneity and *I* ^2^ values in order to evaluate the proportion of observed variance due to variation in real effects rather than sampling error. To assess heterogeneity, the prediction interval for odds ratios will be calculated according to the guidelines of Borenstein et al. (2017).

#### 3.3.10 Assessment of reporting biases

Publication bias and small‐study effects will be addressed through three methods:

1. Small‐study effects will be assessed by constructing funnel plots for each dependent variable (Sterne et al., 2017);
2. Harbord's and Peters' tests will be performed assuming a random‐effects meta‐analysis model. Both tests can be used for two‐sample binary data with log odds‐ratio as effect sizes (Harbord et al., 2016; Peters et al., 2010);
3. If an adequate study sample‐size, substantial homogeneity, and lack of questionable research practices are found in primary studies in accordance with Carter et al. (2019), a three‐parameter selection model will be applied to assess potential publication bias (Carter et al., 2019; Vevea & Woods, 2005).

#### 3.3.11 Data synthesis

All analyses will be performed using STATA 17 (StataCorp). Extracted data will cover the factors associated with early cessation of breastfeeding upon returning to work, categorized according to the Social‐Ecological Model in individual, interpersonal, community, institutional, and public policy factors. Studies with a high (according to RoB 2 or the JBI Critical Appraisal Tools) or critical (according to ROBINS‐I) risk of bias will not be included in the meta‐analysis. Odds ratios will be transformed into the logarithmic scale before using a random‐effects model with a restricted maximum likelihood estimation method to pool the effect sizes. Random‐effect models account for certain study heterogeneity and allow inference for the population of studies based on the sample of studies used in the meta‐analysis; the restricted maximum likelihood method produces an unbiased, nonnegative estimate of the between‐study variability (*τ* ^2^) (Raudenbush, 2009). Given the broad scope of the proposed review questions regarding the multiple possible factors of interest affecting the outcome, a great heterogeneity of study designs and sociodemographic characteristics of participants is expected. Also, studies addressing risk and etiology usually show significant differences in the covariables included in their analyses and might lack sufficient methodological detail (Moola et al., 2015). These factors will be considered to assess the possibility of performing a meta‐analysis.

If meta‐analysis is not possible, a tabular and narrative approach will be used for data reporting (Moola et al., 2020). All reviewers will agree beforehand on a structure for the reporting of results to ensure presentation consistency. Data will be tabulated according to study characteristics and the previously‐mentioned levels of influence, as appropriate. A narrative summary will describe the scope, context, risk of bias, and relevant findings of the studies following the guidelines provided by Popay et al. (2006) and Moola et al. (2020). Also, a discussion on how the findings impact health practices and future research will accompany this synthesis. Finally, existing gaps in published literature will be highlighted to guide future research.

#### 3.3.12 Subgroup analysis and investigation of heterogeneity

If sufficient evidence is found, the following work‐related variables will be used to perform subgroup analysis, given their influence on breastfeeding initiation and duration (Ogbuanu et al., 2011):

- Type of work or job position
- Workers with formal or informal employment
- Dependent or independent (i.e., self‐employed) workers
- Women with full‐time or part‐time employment
- Time since returning to work after birth: <1 month, 1–3 months, >3–<6 months, and ≥6 months.

Subgroup differences will be evaluated by comparing the values of *I* ^2^, performing the test of group differences (*Q_b_*), and analyzing the respective forest plots.

#### 3.3.13 Sensitivity analysis

The robustness of the results will be determined by performing a set of sensitivity analyses:

- First, individual studies will be systematically excluded from the meta‐analysis and the overall effect size and heterogeneity will be recomputed to assess the impact of each study on the overall results.
- Potential outliers will be identified through visual inspection of the forest and funnel plots, and their impact on the study results will be evaluated by excluding them from analyses.
- The effect of different study designs will be examined by separately analyzing randomized controlled trials, nonrandomized intervention studies, cohort studies, case‐control studies, and analytical cross‐sectional studies.
- The methodological quality effect of primary studies will be evaluated by performing separate analyses on studies with a low risk of bias versus studies with some concern, moderate, serious, or unclear risk of bias, as appropriate.

#### 3.3.14 Treatment of qualitative research

We do not plan to include qualitative research.

#### 3.3.15 Summary of findings and assessment of the certainty of the evidence

Evidence body quality will be evaluated using the Grading of Recommendations Assessment, Development, and Evaluation (GRADE) approach for prognosis factors as suggested by Foroutan et al. (2020). This adaptation is adequate for systematic reviews of etiology and risk (Stern et al., 2024). This methodology considers eight domains that potentially impact the evidence certainty: risk of bias, consistency, directness, precision, or publication bias (lowering certainty), and large effect, dose–response, or plausible confounding (enhancing certainty) (Foroutan et al., 2020). The software GRADEpro will be used for this evaluation (GRADEpro GDT, 2024). Finally, a summary‐of‐findings table will include information on the number of participants for each study, odds ratios (and their respective 95% confidence intervals), risk of bias, certainty of evidence, and additional comments in accordance with Schünemann et al. (2023).

## CONTRIBUTIONS OF AUTHORS

- Content: Scotta AV, Barral PE, Farre A, Soria EA
- Systematic review methods: Scotta AV, Barral PE
- Statistical analysis: Scotta AV, Soria EA
- Information retrieval: Scotta AV, Barral PE, Farre A, Soria EA

## DECLARATIONS OF INTEREST

The authors have no potential conflicts of interest to declare.

## SOURCES OF SUPPORT

**Internal sources**

- SECyT, Argentina

Funding was provided by the Secretaría de Ciencia y Técnica at the National University of Córdoba (SECYT‐UNC), grant code: RESOL‐2023‐258‐E‐UNC‐SECYT#ACTIP, 2023–2027.

The funding source played no part in designing or conducting this review.

**External sources**

- No sources of support provided.

## Supporting information

Supporting information.

Supporting information.

Supporting information.

Supporting information.
