# Supplementary material for: Protocol: Factors contributing to the discontinuation of breastfeeding upon women's return to work: A systematic review protocol
Source: Campbell Syst Rev. 2024 Sep 9;20(3):e1434. doi: 10.1002/cl2.1434 (PMC11382015; doi:10.1002/cl2.1434)
Supplement: Supplementary file 1 — Supporting information. [file CL2-20-e1434-s004.docx]

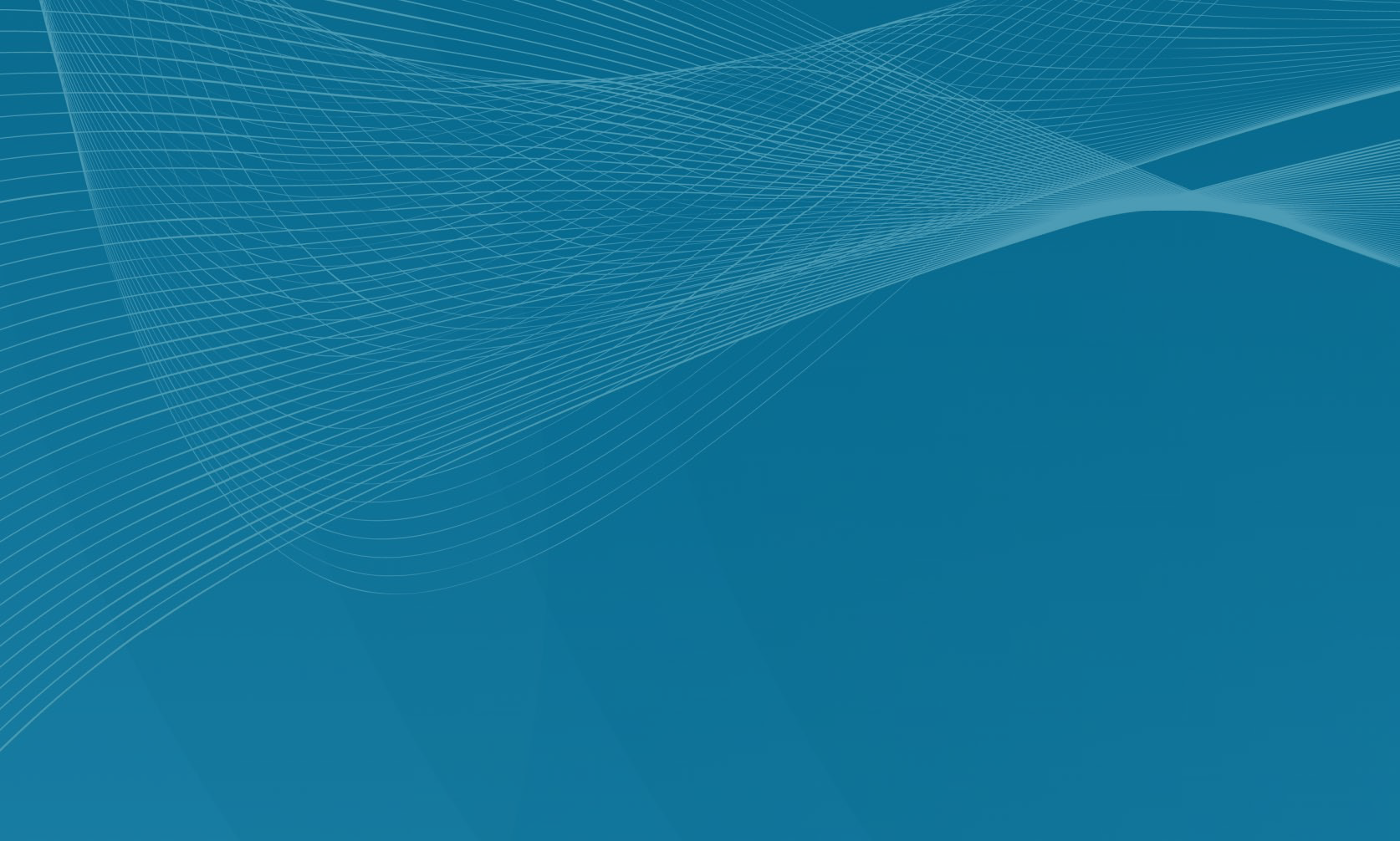


| **Campbell Policies and Guidelines Series No. 3** |
| --- |
| December 2017 |
|  |
|  |
|  |
|  |
|  |
|  |
|  |
|  |
|  |
| **Methodological expectations of Campbell Collaboration intervention reviews: Conduct standards** |


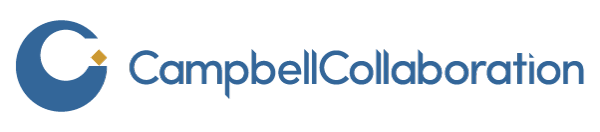


**Colophon**

| **Title** | |  | | Methodological expectations of Campbell Collaboration intervention reviews: Conduct standards |
| --- | --- | --- | --- | --- |
| **Authors** | |  | | The Methods Group of the Campbell Collaboration |
| **DOI** | |  | | 10.4073/cpg.2016.3 |
| **No. of pages** | |  | | 23 |
| **Last updated** | |  | | 6 December 2017 |
| **Citation** | |  | | The Methods Group of the Campbell Collaboration. Methodological expectations of Campbell Collaboration intervention reviews: Conduct standards  Campbell Policies and Guidelines Series No. 3  DOI: 10.4073/cpg.2016.3 |
| **ISSN** | |  | | 2535-2458 |
| **Copyright** | |  | | © The Campbell Collaboration  This is an open-access article distributed under the terms of the Creative Commons Attribution License, which permits unrestricted use, distribution,  and reproduction in any medium, provided the original author and source are credited. |
| **Acknowledgement** | |  | | Adaptations on MECIR Version 2.2 Conduct Standards (Chandler, Churchill, Higgins, Lasserson, & Tovey, 2012) |
| **Editor in Chief** |  | | Vivian Welch, University of Ottawa, Canada | |
| **Chief Executive Officer** |  | | Howard White, The Campbell Collaboration | |
| **Managing Editor** |  | | Chui Hsia Yong, The Campbell Collaboration | |
|  |  | | The Campbell Collaboration was founded on the principle that systematic reviews on the effects of interventions will inform and help improve policy and services. Campbell offers editorial and methodological support to review authors throughout the process of producing a systematic review. A number of Campbell's editors, librarians, methodologists and external peer-reviewers contribute. | |
|  |  | | The Campbell Collaboration  P.O. Box 7004  St. Olavs plass  0130 Oslo, Norway  [www.campbellcollaboration.org](http://www.campbellcollaboration.org) | |
|  | |  | |  |

**Note for authors:**

This document provides detailed methodological expectations for the conduct of Campbell Collaboration systematic reviews of ***intervention effects***. It is important to note that some Campbell reviews may not focus on intervention effects, but may synthesize observational research that is policy relevant. For instance, such reviews may examine correlational or descriptive research, diagnostic or test accuracy, or other topics that do not necessarily focus on intervention effects. Although most of the methodological expectations listed below will be appropriate for all review topics (intervention focused or not), some (particularly those related to study design) may not be entirely applicable to non-intervention reviews, and have been noted as such under the ‘rationale and elaboration’ column.

Status: Mandatory means that a new protocol or review will not be published if this standard is not met. Highly desirable means that this should generally be done but that there are justifiable exceptions. There may be legitimate variation between or within Campbell Coordinating Groups in the relative emphasis placed on compliance with highly desirable standards. The emphasis placed on compliance with highly desirable standards will remain at the discretion of each Campbell Coordinating Group. Optional means this is done at the authors’ discretion.

| **Item No.** | **Status**  **(T = Title,**  **P = Protocol, R = Review)** | **Item Name** | **Standard** | **Rationale and elaboration** | **Relevant Cochrane Handbook section(s)** | **Authors**  **note: Section and para where addressed** |
| --- | --- | --- | --- | --- | --- | --- |
| Setting the research question(s) to inform the scope of the review | | | | | |  |
| C1 | Mandatory  (T & P) | Formulating review questions | Ensure that the review question and particularly the outcomes of interest, address issues that are important to stakeholders such as consumers, practitioners, policy makers, and others. | Campbell reviews are intended to support practice and policy, not just scientific curiosity. The needs of consumers play a central role in Campbell Reviews and they should play an important role in defining the review question | 2.3.2  2.3.4  17.2  20.2.2 | Background, Page 5  Objectives, Page 7 |
| C2 | Mandatory  (T & P) | Pre-defining objectives | Define in advance the objectives of the review, including participants, interventions, comparators, and outcomes. | Objectives give the review focus and must be clear before appropriate eligibility criteria can be developed. If the review will address multiple interventions, clarity is required on how these will be addressed (e.g. summarized separately, combined or explicitly compared). | 5.1.1 | Objectives, Page 7 |
| C3 | Highly desirable  (P) | Considering potential adverse effects | Consider any important potential adverse effects of the intervention(s) and ensure that they are addressed. | It is important that adverse effects are addressed if applicable in order to avoid one-sided summaries of the evidence. In these cases, the review will need to highlight the extent to which potential adverse effects have been evaluated in any included studies. Sometimes data on adverse effects are best obtained from non-randomized studies, or qualitative research studies. This does not mean however that all reviews must include non-randomized studies. | 5.4.3  14.1.1  14.3 | Not applicable. This review is not focused on an intervention. |
| C4 | Highly desirable  (P) | Considering equity and specific populations | Consider in advance whether issues of equity and relevance of evidence to specific populations are important to the review, and plan for appropriate methods to address them if they are. Attention should be paid to the relevance of the review question to populations such as low socioeconomic groups, low or middle-income regions, women, children, people with disabilities, and older people. | Where possible reviews should include explicit descriptions of the effects of the interventions not only on the whole population but also describe their effects upon specific population subgroups and/or their ability to reduce inequalities and to promote their use to the community. |  | Background, Page 5.  Data extraction form, Suppl. Material IV |
| Setting eligibility criteria for including studies in the review | | | | | |  |
| C5 | Mandatory  (P) | Pre-defining unambiguous criteria for participants | Define in advance the eligibility criteria for participants in the studies. | Pre-defined, unambiguous eligibility criteria are a fundamental pre-requisite for a systematic review. The criteria for considering types of people included in studies in a review should be sufficiently broad to encompass the likely diversity of studies, but sufficiently narrow to ensure that a meaningful answer can be obtained when studies are considered in aggregate. Considerations when specifying participants include setting, age, identifying personal characteristics, demographic factors, and other factors that differentiate the participants. Any restrictions to study populations must be based on a sound rationale, since it is important that Campbell reviews are widely relevant. | 5.2 | Methods, page 7 |
| C6 | Highly desirable  (P) | Pre-defining a strategy for studies with a subset of eligible participants | Define in advance how to handle studies in which only a subset of the sample is eligible for inclusion in the review. | Sometimes a study includes some ‘eligible’ participants and some ‘ineligible’ participants, for example when an age cut-off is used in the review’s eligibility criteria. In case data from the eligible participants cannot be retrieved, a mechanism for dealing with this situation should be pre-specified. | 5.2 | Not applicable. The criteria for potential eligible participants are meant to be as broad as possible. |
| C7 | Mandatory  (P) | Pre-defining unambiguous criteria for interventions and comparators | Define in advance the eligible interventions and the interventions against which these can be compared in the included studies. | Pre-defined, unambiguous eligibility criteria are a fundamental pre-requisite for a systematic review. Specification of comparator interventions requires particular clarity, including the extent to which the experimental interventions are compared with a control or comparison conditions with matched or similar participants. Any restrictions on interventions and comparators, such as regarding delivery, dose, duration, intensity, co-interventions, and features of complex interventions should also be pre-defined and explained. | 5.3 | Not applicable.  This review is not focused on an intervention. |
| C8 | Mandatory  (P & R) | Clarifying role of outcomes | Clarify in advance whether outcomes listed under 'Criteria for Inclusion and Exclusion of Studies in the Review’ are used as criteria for including studies (rather than as a list of the outcomes of interest within whichever studies are included). | Outcome measures need not always form part of the criteria for including studies in a review. However, some reviews do legitimately restrict eligibility to specific outcomes. For example, the same intervention may be studied in the same population for different purposes (e.g. reading interventions); or a review may address specifically the adverse effects of an intervention used for several conditions. If authors do exclude studies on the basis of outcomes, care should be taken to ascertain that relevant outcomes are not available because they have not been measured rather than simply not reported. | 5.1.2 | Methods, page 8, subheading “Types of outcome measures”. |
| C9 | Mandatory  (P) | Pre-defining study designs | Define in advance the eligibility criteria for study designs in a clear and unambiguous way, with a focus on features of a study's design rather than design labels. For reviews with multiple objectives, specify whether study design inclusion criteria are common across all questions, or identified separately for each type of question. | Pre-defined, unambiguous eligibility criteria are a fundamental pre-requisite for a systematic review. This is particularly important when non-randomized (e.g., quasi-experimental or observational) studies are considered. Some labels commonly used to define study designs can be ambiguous. For example a "double blind" study may not make it clear who is blind; a "case control" study may be nested within a cohort, or be undertaken in a cross- sectional manner; or a "prospective" study may have only some features defined or undertaken prospectively. | 5.5  13.2.2 | Methods, page 8, subheading “Types of studies”.  Data extraction form, Suppl. Material IV |
| C10 | Mandatory  (P, effectiveness reviews only) | Including randomized trials | Include randomized trials as eligible for inclusion in the review, if they are feasible and available for the interventions, outcomes, and populations of interest. | Randomized trials are the best study design for evaluating the efficacy of many interventions. If they are feasible for evaluating questions that are being addressed by the review, they must be considered eligible for the review. However, appropriate exclusion criteria may be put in place, for example regarding length of follow-up. | 5.5  13.1.3 | Methods, page 8, subheading “Types of studies”.  Data extraction form, Suppl. Material IV |
| C11 | Mandatory  (P) | Justifying choice of study designs | Justify the choice of eligible study designs. | The particular study designs included should be justified with regard to appropriateness to the review question and with regard to potential for bias. It might be difficult to address some interventions or some outcomes in randomized trials. Authors should be able to justify why they have chosen either to restrict the review to randomized trials or to include non-randomized studies. | 13.1.2  13.2.1.3 | Methods, page 8, subheading “Types of studies”. |
| C12 | Mandatory  (P & R) | Including studies regardless of publication status | Include studies irrespective of their publication status, and their electronic availability. | Obtaining and including data from unpublished studies (including grey literature) can reduce the effects of publication bias. | 6.2.3  10.3.2 | Methods, Page 8-10, subheadings “Types of studies” and “Selection of studies”. |
| C13 | Mandatory  (R) | Changing eligibility criteria | Justify any changes to eligibility criteria or outcomes studied. In particular, *post hoc* decisions about inclusion or exclusion of studies should keep faith with the objectives of the review rather than with arbitrary rules. | Following pre specified eligibility criteria is a fundamental attribute of a systematic review. However unanticipated issues may arise. Review authors should make sensible *post hoc* decisions about exclusion of studies, and these should be documented in the review, possibly accompanied by sensitivity analyses. Changes to the protocol must not be based on findings of the studies or the synthesis, as this can introduce bias. | 5.2  5.7 | Not applicable. This is a review protocol. |
| Selecting outcomes to be addressed for studies included in the review | | | | | |  |
| C14 | Mandatory  (P) | Pre-defining outcomes | Define in advance which outcomes are primary outcomes and which are secondary outcomes. | Pre-definition of outcome reduces the risk of selective outcome reporting. The *primary outcomes* should be as few as possible (ideally no more than three). It is expected that the review should be able to synthesize these outcomes if eligible studies are identified, and that the conclusions of the review will be based in large part on the effect of the interventions on these outcomes. | 5.4.2 | Methods, page 8, subheading “Primary outcomes” |
| C15 | Highly desirable  (P) | Choosing outcomes | Keep the total number of outcomes selected for inclusion in the review as small as possible. Choose outcomes that are relevant to stakeholders such as consumers, practitioners, and policy makers. Consider the importance of resource use and cost outcomes. | Campbell reviews are intended to support practice and policy, and should address outcomes that are important to consumers. These should be specified at protocol stage. Where they are available, established sets of core outcomes should be used. Participant-reported outcomes should be included where possible. It is also important to judge whether evidence on resource use and costs might be an important component of decisions to adopt the intervention or alternative management strategies around the world. Large numbers of outcomes, while sometimes necessary, can make reviews unfocused, unmanageable for the user, and prone to selective outcome reporting bias. | 5.4.2 | Methods, page 8, subheading “Types of outcome measures”. |
| C16 | Highly desirable  (P) | Pre-defining outcome details | Define in advance details of what are acceptable outcome measures (e.g., test scores conditions, characteristics, scales, composite outcomes). | Having decided what outcomes are of interest to the review, authors should clarify acceptable ways in which these outcomes can be measured. | 5.4.1 | Methods, page 8, subheading “Types of outcome measures”. |
| C17 | Highly desirable  (P) | Pre-defining choices from multiple outcome measures | Define in advance how outcome measures will be selected at the coding stage when there are several possible measures (e.g. multiple definitions, assessors, or scales) or at the analysis stage if multiple effect sizes are coded per outcome construct. | Pre-specification guards against selective outcome reporting or selective analysis, and allows users to confirm that choices were not overly influenced by the results. A pre-defined hierarchy of outcome measures may be helpful. It may however be difficult to pre- define outcome measures for adverse effects. A rationale should be provided for the choice of all outcome measures (including adverse effects). | 5.4.1 | Not applicable. The outcome “early breastfeeding cessation” is dichotomic. |
| C18 | Highly desirable  (P) | Pre-defining time points of interest | Define in advance how differences in the timing of outcome measurement will be handled in the review. | Pre-specification guards against selective outcome reporting or selective analysis, and allows users to confirm that choices were not overly influenced by the results. Authors may consider whether all time frames or only selected time-points will be included in the review. These decisions should be based on outcomes important for making policy or practice decisions. One strategy to make use of the available data could be to group time- points into pre-specified intervals to represent ‘short-term’, ‘medium-term’, and ‘long- term’ outcomes and to use information on no more than one from each interval from each study for any particular outcome. | 5.4.1 | Methods, page 9, subheading “Duration of follow-up”. |
| Planning the review methods at protocol stage | | | | | |  |
| C19 | Mandatory  (P) | Planning the search | Plan in advance the methods to be used for identifying studies. Design searches to capture as many studies as possible meeting the eligibility criteria, ensuring that relevant time periods and sources are covered and not restricting by language or publication status. | Searches should be motivated directly by the eligibility criteria for the review, and it is important that all types of eligible studies are considered when planning the search. There is a possibility of publication bias and/or language bias (whereby the language of publication is selected in a way that depends on the findings of the study) if searches are restricted by publication status or by language of publication. Removing language restrictions in English-language databases is not a good substitute for searching non-English language journals and databases. | 6.3  6.4 | Methods, page 9, subheading “Search methods for identification of studies”.  Suppl. Material III |
| C20 | Mandatory  (P) | Planning the assessment of risk of bias/study quality in the included studies | Plan in advance the methods to be used for assessing risk of bias/study quality in included studies, including the tool(s) or codes to be used, how the tool(s) or codes will be implemented, and the criteria used to assign studies to risk of bias or quality categories (at outcome- and/or study-level), for example, low risk, high risk, and unclear risk of bias; low quality or high quality. | Pre-defining the methods and criteria for assessing risk of bias/study quality is important because analysis or interpretation of the review findings may be affected by the judgments made during this process. For randomized trials, the Cochrane risk of bias tool is a recommended option. | 8.3 | Methods, page 11, subheading “Assessment of risk of bias in included studies”. |
| 21 | Mandatory  (P) | Planning the synthesis of results | Plan in advance the methods to be used to synthesize the results of the included studies, including whether a quantitative synthesis is planned, how heterogeneity will be assessed, choice of effect measure (e.g., standardized mean difference, odds ratio, risk ratio), and methods for meta-analysis (e.g. inverse variance or Mantel Haenszel, fixed-effect or random- effects model). If a quantitative synthesis is not planned, or if it is not possible, plan the specific methods to narratively synthesize the results of the included studies. | Pre-defining the synthesis methods, particularly the statistical methods, is important because analysis or interpretation of the review findings may be affected by the judgments made during this process. | 9.1.2 | Methods, page 13, subheading “Data synthesis”. |
| C22 | Mandatory  (P) | Planning moderator analyses | Pre-define potential effect modifiers for moderator analyses (e.g. subgroup analyses or meta-regression analyses) at the protocol stage; restrict these in number; and provide rationale for each. | Pre-specification reduces the risk that large numbers of undirected moderator analyses lead to spurious explanations of heterogeneity | 9.6.5 | Methods. page 14, subheading “Subgroup analysis and investigation of heterogeneity” |
| C23 | Optional  (P) | Planning a ‘Summary of findings’ table | If a formal ‘Summary of findings’ table is anticipated, specify which outcomes will be included, and which comparisons and subgroups will be covered (if appropriate). | The ‘Summary of findings table’ offers a specific approach to summarizing the findings of a systematic review of intervention effects. Its use is not mandatory or recommended in Campbell Reviews of intervention effects but is highly desirable if the review is co-registered with a Cochrane group. Methods for ‘Summary of findings’ tables should be pre-defined, particularly with regard to choice of outcomes, to guard against selective presentation of results in the review.  If included, the table should include the essential outcomes for decision making (typically up to seven), which should generally not include surrogate or interim outcomes. These outcomes should not be chosen on the basis of any anticipated or observed magnitude of effect, or because they are likely to have been addressed in the studies to be reviewed. Outcome-level summary risk of bias judgments made using the Cochrane Risk of Bias tool feed directly into the ‘Study limitations’ column of a formal ‘Summary of findings table’. Therefore, authors planning a formal ‘Summary of findings table’ should plan to use the Cochrane Risk of Bias tool in their assessments of risk of bias. | 11.5 | Methods, page 14-15, subheading “Summary of findings and assessment of the certainty of the evidence” |
| C24 | Mandatory  (P) | Planning the search | Refer to “Searching for Studies”, the Campbell information retrieval guide, to ensure that all relevant databases have been properly searched. | Searches for studies should be as extensive as possible to reduce the risk of publication bias and to identify as much relevant evidence as possible. There is no minimum set of databases to search, but reviewers should consider consulting with a research retrieval specialist to avoid unnecessary duplication of effort. | 6.2.1.1  6.3.3 | Methods, page 9, subheading “Search methods for identification of studies”. |
| C25 | Highly desirable  (P) | Searching specialist bibliographic databases | Search appropriate national, regional, and subject specific bibliographic databases. | Searches for studies should be as extensive as possible to reduce the risk of publication bias and to identify as much relevant evidence as possible. Databases relevant to the review topic should be covered (e.g., ERIC for educational interventions, PsycINFO for psychological interventions), and regional databases (e.g. LILACS) should be considered. | 6.2.1.4  6.2.1.5  6.4.1 | Methods, page 9, subheading “Electronic searches”. |
| C26 | Mandatory (if applicable)  (P) | Searching for different types of evidence | If the review has specific eligibility criteria around study design to address adverse effects, economic issues, or qualitative research questions, undertake searches to address them. | Sometimes different searches will be conducted for different types of evidence, such as for non-randomized studies for addressing adverse effects, or for economic evaluation studies. | 13.3  14.5  15.3  20.3.2.1 | Not applicable. Given the wide range of sources and a clear outcome definition, specific criteria are not required. |
| C27 | Mandatory (if applicable)  (P) | Searching trials registers | When relevant, search trials registers and repositories of results, where relevant to the topic through ClinicalTrials.gov, metaREGISTER, the WHO International Clinical Trials Registry Platform (ICTRP) portal, and other sources as appropriate. | When relevant, searches for studies should be as extensive as possible to reduce the risk of publication bias and to identify as much relevant evidence as possible. Although ClinicalTrials.gov is included as one of the registers within the WHO ICTRP portal, it is recommended that both ClinicalTrials.gov and the ICTRP portal are searched separately due to additional features in ClinicalTrials.gov. | 6.2.3.1  6.2.3.2  6.2.3.3 | Methods, page 10, subheading “Searching other resources”. |
| C28 | Mandatory  (P) | Searching for grey literature | Search relevant grey literature sources such as reports/dissertations/theses databases and databases of conference abstracts. | Searches for studies should be as extensive as possible to reduce the risk of publication bias and to identify as much relevant evidence as possible. | 6.2.1.7  6.2.1.8  6.2.2 | Methods, page 10, subheading “Searching other resources”. |
| C29 | Mandatory  (P) | Searching within other reviews | Search within previous reviews on the same or similar topic. | Searches for studies should be as extensive as possible to reduce the risk of publication bias and to identify as much relevant evidence as possible. | 6.2.2.5 | Methods, page 8, subheading “Types of studies”. |
| C30 | Mandatory  (P) | Searching reference lists | Check reference lists in included studies and any relevant systematic reviews identified. | Searches for studies should be as extensive as possible to reduce the risk of publication bias and to identify as much relevant evidence as possible. | 6.2.2.5 | Methods, page 8, subheading “Types of studies”. |
| C31 | Highly desirable  (P) | Searching by contacting relevant individuals and organizations | Contact relevant individuals and organizations for information about unpublished or ongoing studies. | Searches for studies should be as extensive as possible to reduce the risk of publication bias and to identify as much relevant evidence as possible. It is important to identify ongoing studies, so that when a review is later updated these can be assessed for possible inclusion. | 6.2.3 | Not applicable. |
| C32 | Mandatory  (R) | Structuring search strategies for bibliographic databases | Inform the structure of search strategies in bibliographic databases around the main concepts of the review, using appropriate elements from PICO and study design. In structuring the search, maximize sensitivity whilst striving for reasonable precision. Ensure correct use of the AND and OR operators. | Inappropriate or inadequate search strategies may fail to identify records that are included in bibliographic databases. Expertise may need to be sought, in particular from an Information Retrieval Specialist. The structure of a search strategy should be based on the main concepts being examined in a review. In electronic bibliographic databases, a search strategy to identify studies for a Campbell Review will typically have three sets of terms: 1) terms to search for the population of interest; 2) terms to search for the intervention(s) evaluated; and 3) terms to search for the types of study designs to be included. There are exceptions, however.  For instance, for reviews of complex interventions, it may be necessary to search only for the population or the intervention. Within each concept, terms are joined together with the Boolean ‘OR’ operator, and the concepts are combined with the Boolean ‘AND’ operator. The ‘NOT’ operator should be avoided where possible to avoid the danger of inadvertently removing from the search set records that are relevant. | 6.4.2  6.4.4  6.4.7 | Suppl. Material III. |
| C33 | Mandatory  (R) | Developing search strategies for bibliographic databases | Identify appropriate controlled vocabulary (e.g. MeSH, Emtree, including 'exploded' terms) and free-text terms (considering, for example, spelling variants, synonyms, acronyms, truncation, and proximity operators), and tailor the search strategy to each specific database. | Inappropriate or inadequate search strategies may fail to identify records that are included in bibliographic databases. Search strategies need to be customized for each database. It is important that MeSH terms are ‘exploded’ wherever appropriate, in order not to miss relevant articles. The same principle applies to EMTREE when searching EMBASE and also to a number of other databases. The controlled vocabulary search terms are different for each electronic database, and thus search strategies must be tailored to each database. To be as comprehensive as possible, it is necessary to include a wide range of free-text terms for each of the concepts selected. This might include the use of truncation and wildcards. Developing a search strategy is an iterative process in which the terms that are used are modified, based on what has already been retrieved. | 6.4.5  6.4.6  6.4.8 | Not applicable. This is a review protocol. |
| C34 | Highly desirable  (R) | Using search filters | Use specially designed and tested search filters where appropriate (such as the Cochrane Highly Sensitive Search Strategies for identifying randomized trials in MEDLINE), but do not use filters in pre- filtered databases (e.g. do not use a randomized trial filter in CENTRAL or a systematic review filter in DARE or PROSPERO). | Search filters should be used with caution. They should be assessed not only for the reliability of their development and reported performance but also for their current accuracy, relevance, and effectiveness given the frequent interface and indexing changes affecting databases. | 6.4.11  6.4.2  13.3.1.2  14.5.2  15.3.1  17.5  20.3.2.1 | Not applicable. This is a review protocol. |
| C35 | Mandatory  (P & R) | Restricting database searches | Justify the use of any restrictions in the search strategy on publication date, publication format, or language. | Date restrictions in the search should only be used when there are date restrictions in the eligibility criteria for studies. They should be applied only if it is known that relevant studies could only have been reported during a specific time period, for example if the intervention was only available after a certain time point. Searches for updates to reviews might naturally be restricted by date of entry into the database (rather than date of publication) to avoid duplication of effort. Publication format restrictions (e.g. exclusion of letters) should generally not be used in Campbell reviews, since any information about an eligible study may be of value. | 6.4.9 | Methods, page 9, subheading “Search methods for identification of studies”. |
| C36 | Mandatory  (P & R) | Documenting the search process | Document the search process in enough detail to ensure that it can be reported correctly in the review/ update. Include the month and year the search began and ended for future replicability. | The search process (including the sources searched, when, by whom, and using what terms) needs to be documented in enough detail throughout the process to ensure that it can be reported correctly in the review, to the extent that all the searches of all the databases are reproducible. | 6.6.1 | Methods, page 9, subheading “Search methods for identification of studies”. |
| C37 | Highly desirable  (R) | Rerunning searches | Rerun or update searches for all relevant databases within 12 months before publication of the review or review update, and screen the results for potentially eligible studies. | The published review should be as up to date as possible. The search should be rerun close to publication, if the initial search date is more than 12 months (preferably 6 months) from the intended publication date, and the results screened for potentially eligible studies. Ideally the studies should be fully incorporated. If not, then the potentially eligible studies will need to be reported, at a minimum as a reference under ‘Studies awaiting classification’ or ‘Ongoing studies’. |  | Not applicable. This is a review protocol. |
| C38 | Highly desirable  (R) | Incorporating findings from rerun searches | Incorporate fully any studies identified in the rerun or update of the search within 12 months before publication of the review or review update. | The published review should be as up to date as possible. After the rerun of the search, the decision whether to incorporate any new studies fully into the review will need to be balanced against the delay in publication. |  | Not applicable. This is a review protocol. |
| Selecting studies into the review | | | | | |  |
| C39 | Highly desirable  (P & R) | Making inclusion decisions in duplicate | The preferred procedure is for at least two members of the review team to independently screen candidate studies and resolve discrepancies by consensus. Where large numbers of studies are involved, samples of the candidate studies might be drawn and rescreened to estimate the reliability of the inclusion decisions. | Duplicating the study selection process reduces both the risk of making mistakes and the possibility that selection is influenced by a single person’s biases. The inclusion decisions should be based on the full texts of potentially eligible studies when possible, usually after an initial screen of titles and abstracts. It is desirable, *but not mandatory*, that two people undertake this initial screening, working independently. | 7.2.4 | Methods, page 10, subheading “Selection of studies”. |
| C40 | Mandatory  (P & R) | Including studies without useable data | Include studies in the review irrespective of whether measured outcome data are reported in a ‘usable’ way. | Systematic reviews typically should seek to include all relevant participants who have been included in eligible study designs of the relevant interventions and had the outcomes of interest measured. Reviews must not exclude studies solely on the basis of *reporting* of the outcome data, since this may introduce bias due to selective outcome reporting (i.e., that an effect size is not estimable although the outcome was clearly measured). While such studies cannot be included in meta- analyses, the implications of their omission should be considered. Note that studies may legitimately be excluded because outcomes were not *measured*. Furthermore, issues may be different for adverse effects outcomes, since the pool of studies may be much larger and it can be difficult to assess whether such outcomes were measured. | 5.4.1 | Methods, page 12, subheading  “Unit of analysis issues”. |
| C41 | Mandatory  (R) | Documenting decisions about records identified | Document the selection process in sufficient detail to complete a PRISMA flow chart and a table of ‘Characteristics of excluded studies’. | A PRISMA flow chart and a table of ‘Characteristics of excluded studies’ will need to be completed in the final review. Decisions should therefore be documented for all records identified by the search. Numbers of records are sufficient for exclusions based on initial screening of titles and abstracts. Broad categorizations are sufficient for records classed as potentially eligible during an initial screen. Studies listed in the table of ‘Characteristics of excluded studies’ should be those which a user might reasonably expect to find in the review. At least one explicit reason for their exclusion must be documented. Authors will need to decide for each review when to map records to studies (if multiple records refer to one study). Lists of included and excluded studies must be based on studies rather than records. | 6.6.1*  11.2.1* | Not applicable. This is a review protocol. |
| C42 | Mandatory  (R) | Collating multiple reports | Collate multiple reports of the same study, so that each study rather than each report is the unit of interest in the review. | It is wrong to treat multiple reports of the same study as if they are multiple studies. Secondary reports of a study should not be discarded, however, since they may contain valuable information about the design and conduct. Review authors must choose and justify which report to use as a source for study results. | 7.2.1  7.2.2  7.6.4 | Not applicable. This is a review protocol. |
| C43 | Mandatory  (P & R) | Using data collection forms | Use a data collection form, which has been piloted. | Review authors often have different backgrounds and level of systematic review experience. Using a data collection form ensures some consistency in the process of data extraction, and is helpful if comparing data extracted in duplicate. The original data collection forms should be included in the protocol for the review. If the data collection forms are altered during pilot testing, the final data collection forms should be submitted in an appendix with the final review. | 7.5 | Suppl. Material III. |
| C44 | Mandatory  (R) | Describing studies | Collect characteristics of the included studies in sufficient detail to populate final tables and narrative overview. | Basic characteristics of each study will need to be presented as part of the review, including details of participants, interventions and comparators, outcomes and study design. | 7.3  11.2 | Not applicable. This article is a review protocol. |
| C45 | Highly desirable  (P & R) | Extracting study characteristics and outcome data in duplicate | The preferred procedure is for at least two members of the review team to independently code each study and resolve any discrepancies through discussion and consensus. Where large number of studies makes this procedure too demanding, random samples of the studies can be drawn and recoded by a different team member so that the reliability of the coding can be assessed and reported. The procedures planned for training coders and checking their accuracy before they begin providing data for the review should also be described along with the relevant background of those expected to do the coding. | Duplicating the data extraction process reduces both the risk of making mistakes and the possibility that data selection is influenced by a single person’s biases. Dual data extraction is particularly important for outcome data, which feed directly into syntheses of the evidence and hence to conclusions of the review. | 7.6.2  7.6.5 | Methods, page 11, subheading “Data extraction and management”. |
| C46 | Mandatory  (P & R) | Making maximal use of data | Collect and utilize the most detailed numerical data that might facilitate similar analyses of included studies. Where 2×2 tables or means and standard deviations are not available, this might include effect estimates (e.g. odds ratios, regression coefficients), confidence intervals, test statistics (e.g. t, F, Z, chi-squared), p-values, or even data for individual participants. | Data entry into most specialized computer software for meta-analysis is easiest when 2×2 tables are reported for dichotomous outcomes or when means and standard deviations are presented for continuous outcomes. Sometimes these statistics are not reported but some manipulations of the reported data can be performed to obtain them. For instance, 2×2 tables can often be derived from sample sizes and percentages, while standard deviations can often be computed using confidence intervals or p-values. Multiple software options are available for conversions. | 7.7 | Methods, page 11, subheading “Measures of treatment effect” |
| C47 | Highly desirable  (R) | Examining errata | Examine any relevant retraction statements and errata for information. | Some studies may have been found to be fraudulent or may for other reasons have been retracted since publication. Errata can reveal important limitations, or even fatal flaws, in included studies. All of these may potentially lead to the exclusion of a study from a review or meta-analysis. Care should be taken to ensure that this information is retrieved in all database searches by downloading the appropriate fields together with the citation data. | 6.4.10 | Not applicable. This article is a review protocol. |
| C48 | Highly desirable  (P & R) | Obtaining unpublished data | Seek key unpublished information that is missing from reports of included studies. | Contacting study authors to obtain or confirm data makes the review more complete, potentially enhancing precision and reducing the impact of reporting biases. Missing information includes details to inform risk of bias/study quality assessments, details of interventions and outcomes, and study results (including breakdowns of results by important subgroups). | 7.4.2 | Methods, page 11, subheading “Data extraction and management”. |
| C49 | Mandatory  (P & R) | Choosing intervention groups in multi-arm studies | If a study is included with more than two intervention arms, include in the review only intervention and control groups that meet the eligibility criteria. | There is no point including irrelevant intervention groups in the review. Authors should however make it clear in the ‘Table of characteristics of included studies’ that these intervention groups were present in the study. | 16.5.2 | Suppl. Material IV. |
| C50 | Mandatory  (R) | Checking accuracy of numeric data in the review | Compare magnitude and direction of effects reported by studies with how they are presented in the review, taking account of legitimate differences. | This is a reasonably straightforward way for authors to check a number of potential problems, including typographical errors in studies’ reports, accuracy of data collection and manipulation, and data entry into a computer software program. For example, the direction of a standardized mean difference may accidentally be wrong in the review. A basic check is to ensure the same qualitative findings (e.g. direction of effect and statistical significance) between the data as presented in the review and the data as available from the original study. Results in forest plots should agree with data in the original report (point estimate and confidence interval) if the same effect measure and statistical model is used. |  | Not applicable. This article is a review protocol. |
| Assessing risk of bias/study quality in included studies | | | | | |  |
| C51 | Mandatory  (P & R) | Assessing risk of bias/study quality | Assess the risk of bias/study quality for each included study, regardless of the study design or randomization type. | Assessing risk of bias/study quality is an important task because it has been shown that risk of bias/study quality can influence estimates of intervention effects. If the review is co-registered and uses randomized controlled trials, then the Cochrane Risk of Bias tool should be used. If not, then one of the many other study quality tools and/or coding schemes for study quality should be utilized and detailed within the protocol prior to implementation.  Coding schemes for study quality are often used in addition to (or instead of) risk of bias/study quality tools in order to code specific quality variables relating to each source of bias/ dimension of study quality.  Campbell reviews should not use composite scales, indices, or other measures that conflate multiple measures of risk of bias/study quality into a single score (e.g., using an average scale that combines measures of allocation concealment, attrition, and baseline equivalence measures). These composite quality scales can be misleading and should not be used in a Campbell review. Instead, any risk of bias/study quality coding should isolate unique measures of quality (e.g. separate measures for allocation concealment, attrition, spillover, selective outcome reporting, selective analysis reporting, and baseline equivalence). | 8.5  8.9  8.10  8.11  8.12  8.13  8.14  8.15* | Methods, page 11, subheading “Assessment of risk of bias in included studies”. |
| C52 | Highly desirable  (P & R) | Assessing risk of bias /study quality in duplicate | Use (at least) two people working independently to apply a risk of bias/study quality tool or coding scheme to each included study, and define in advance the process for resolving disagreements. | Duplicating risk of bias/study quality assessment/ coding reduces both the risk of making mistakes and the possibility that assessments are influenced by a single person’s biases. | 7.6.2  8.3.4 | Methods, page 11, subheading “Assessment of risk of bias in included studies”. |
| C53 | Highly desirable  (R) | Supporting judgments of risk of bias/study quality | If applicable, justify categorical risk of bias/study quality judgments (e.g., high, low, and unclear) with information directly from the study. | Providing support for the judgment makes the process transparent. | 8.5.1  8.5.2 | Not applicable. This article is a review protocol. |
| C54 | Highly desirable  (R) | Providing sources of information for risk of bias/study quality assessments | If applicable, collect the source of information for each risk of bias/study quality assessment. Where judgments are based on assumptions made on the basis of information provided outside publicly available documents, this should be stated. | Readers/editors/referees should have the opportunity to see for themselves where supports for judgments have been obtained. | 8.5.2 | Not applicable. This article is a review protocol. |
| C55 | Highly desirable  (P & R) | Differentiating between performance bias and detection bias | Consider separately the risks of bias due to lack of blinding for (i) participants and study personnel (performance bias), and (ii) outcome assessment (detection bias). | The use of mutually exclusive domains of bias (e.g. selection bias, performance bias, detection bias, attrition bias and reporting bias) provides a more comprehensive framework for considering biases in randomized trials. | 8.5.1  8.11.1*  8.12.1* | Methods, page 11, subheading “Assessment of risk of bias in included studies”. |
| C56 | Only if applicable  (R) | If applicable, assessing risk of bias due to lack of blinding for different outcomes | Consider blinding separately for different key outcomes. | The risk of bias due to lack of blinding may be different for different outcomes. When there are multiple outcomes, they should be grouped (e.g. objective versus subjective). | 8.5.1  8.11.2  8.12.2* | Not applicable. This article is a review protocol. |
| C57 | Only if applicable  (R) | If applicable, assessing completeness of data for different outcomes | Consider the impact of missing data separately for different key outcomes to which an included study contributes data. | When considering risk of bias due to incomplete (missing) outcome data, this often cannot reliably be done for the study as a whole. The risk of bias due to missing outcome data may be different for different outcomes. For example, there may be less drop-out for a three-month outcome than for a six-year outcome. When there are multiple outcomes, they should be grouped (e.g. short term versus long term). Judgments should be attempted about which outcomes are thought to be at high or low risk of bias. | 8.5.1 | Not applicable. This article is a review protocol. |
| C58 | Only if applicable  (R) | If applicable, summarizing risk of bias assessments when using the Cochrane Risk of Bias tool | Summarize the risk of bias for each key outcome for each study. | This reinforces the link between the characteristics of the study design and their possible impact on the results of the study, and is an important pre-requisite for the GRADE approach to assessing the quality of the body of evidence. | 8.7 | Not applicable. This article is a review protocol. |
| C59 | Highly desirable  (R) | Addressing risk of bias/study quality in the synthesis | Address risk of bias/study quality in the synthesis (whether qualitative or quantitative). For example, present analyses stratified according to key risk of bias/study quality items, or conduct a moderator analysis with one or more risk of bias/study quality ratings. | Review authors should consider how study biases affect conclusions. This is useful in determining the strength of conclusions and how future research should be designed and conducted. | 8.8 | Not applicable. This article is a review protocol. |
| C60 | Highly desirable  (R) | Incorporating assessments of risk of bias | If randomized trials have been assessed using one or more tools in addition to the Cochrane ‘Risk of bias’ tool, use the Cochrane tool as the primary assessment of bias for interpreting results, choosing the primary analysis, and drawing conclusions. | For consistency of approach across Campbell reviews, the Cochrane risk of bias tool should take precedence when two or more tools are used. | 8.5 | Not applicable. This article is a review protocol. |
| Synthesizing the results of included studies | | | | | |  |
| C61 | Mandatory  (R) | Combining different scales | If studies are combined with different scales, ensure that higher scores for continuous outcomes all have the same meaning for any particular outcome; explain the direction of interpretation; and report when directions were reversed. | Sometimes scales have higher scores that reflect a ‘better’ outcome and sometimes lower scores reflect ‘better’ outcome. Meaningless (and misleading) results arise when effect estimates with opposite clinical meanings are combined | 9.2.3.2 | Not applicable. This article is a review protocol. |
| C62 | Mandatory  (R) | Ensuring meta-analyses are meaningful | Undertake (or display) a meta-analysis only if participants, interventions, comparisons and outcomes are judged to be sufficiently similar to ensure an answer that is meaningful for the review question. | A single mean effect size from a meta-analysis of a very diverse collection of studies can be misleading. Variability in the nature of the treatment, control/comparison condition, sample characteristics, and intervention context, may be related to observed effects and a single mean effect size may misrepresent that diversity. Diversity does not necessarily indicate that a meta-analysis should not be performed. However, authors must be clear about the underlying question that all studies are addressing and interpret the results appropriately. The determination of whether a meta-analysis is meaningful should be made based on substantive knowledge of the effect sizes being synthesized; it should never be made based on statistical results for heterogeneity assessments. | 9.1.4 | Not applicable. This article is a review protocol. |
| C63 | Mandatory  (P & R) | Assessing statistical heterogeneity | Assess the presence and extent of between-study variation when undertaking a meta-analysis. | The presence of heterogeneity affects the extent to which generalizable conclusions can be formed. It is important to identify heterogeneity in case there is sufficient information to explain it and offer new insights. Authors should recognize that there is much uncertainty in measures such as I-squared and tau-squared when there are few studies. Thus, use of simple thresholds to diagnose heterogeneity should be avoided. | 9.5.2 | Methods, page 13, subheading “Assessment of heterogeneity”. |
| C64 | Highly desirable  (R) | Addressing missing outcome data | Consider the implications of missing outcome data from individual participants (due to losses to follow up or exclusions from analysis). | Incomplete outcome data can introduce bias. In most circumstances, authors should follow the principles of intention to treat analyses as far as possible (this may not be appropriate for adverse effects or if trying to demonstrate equivalence). Imputation methods can be considered (accompanied by, or in the form of, sensitivity analyses). | 16.2 | Not applicable. This article is a review protocol. |
| C65 | Highly desirable  (R) | Addressing skewed data | Consider the possibility and implications of skewed data when analyzing continuous outcomes. | Skewed data are sometimes not usefully summarized by means and standard deviations. While statistical methods are approximately valid for large sample sizes, skewed outcome data can lead to misleading results when studies are small. | 9.4.5.3 | Not applicable. This article is a review protocol. |
| C66 | Mandatory  (P & R) | Addressing studies with more than two groups | If multi-arm studies are included, analyze multiple intervention groups in an appropriate way that avoids arbitrary omission of relevant groups and double- counting of participants. | Excluding relevant groups decreases precision and double counting increases precision spuriously; both are inappropriate and unnecessary. Alternative strategies include combining intervention groups, separating comparisons into different forest plots and using multiple treatments meta-analysis. | 7.7.3.8  16.5.4 | Methods, page 12, subheading “Unit of analysis issues”. |
| C67 | Mandatory  (P & R) | Comparing subgroups | If subgroup analyses are to be compared, and there are judged to be sufficient studies to do this meaningfully, use a formal statistical test to compare them. | Concluding that there is a difference in effect across subgroups based on differences in the level of statistical significance within subgroups can be very misleading. Two groups may have similar treatment effects yet one may be statistically significant and the other not. Any conclusion that the intervention is effective for one group and not for the other should be based on a direct test of the mean difference between the groups (e.g., with meta-analytic analog-to-the-ANOVA or meta-regression). | 9.6.3.1 | Methods, page 14, subheading “Subgroup analysis and investigation of heterogeneity” |
| C68 | Mandatory  (P & R) | Interpreting subgroup analyses | If subgroup analyses are conducted, follow the subgroup analysis plan specified in the protocol without undue emphasis on particular findings. If post hoc subgroup analyses are conducted that were not specified in the protocol, the review must clearly state that these analyses are post hoc and exploratory in nature. | Selective reporting, or over-interpretation, of particular subgroups or particular subgroup analyses should be avoided. This is especially a problem when multiple subgroup analyses are performed. This does not preclude the use of sensible and honest post hoc subgroup analyses. | 9.6.5.2 | Methods, page 14, subheading “Subgroup analysis and investigation of heterogeneity” |
| C69 | Mandatory  (R) | Considering statistical heterogeneity when interpreting the results | Take into account any statistical heterogeneity when interpreting the results, particularly when there is variation in the direction of effect. | The presence of heterogeneity affects the extent to which generalizable conclusions can be formed. If a fixed-effect analysis is used, the confidence intervals ignore the extent of heterogeneity. If a random-effects analysis is used, the result pertains to the mean effect across studies. In both cases, the implications of notable heterogeneity should be addressed. It may be possible to understand the reasons for the heterogeneity if there are sufficient studies. | 9.5.4 | Not applicable. This article is a review protocol. |
| C70 | Mandatory  (P & R) | Addressing non- standard designs | Consider the impact on the analysis of clustering, matching, or other non-standard design features of the included studies. | Cluster-randomized trials, cross-over trials, studies involving measurements on multiple body parts, and other designs need to be addressed specifically, since a naive analysis might underestimate or overestimate the precision of the study. Failure to account for clustering is likely to overestimate the precision of the study - i.e. to give it confidence intervals that are too narrow and a weight that is too large. Failure to account for correlation is likely to underestimate the precision of the study, i.e., to give it confidence intervals that are too wide and a weight that is too small. | 9.3  16.3  16.4 | Methods, page 12, subheading “Unit of analysis issues” |
| C71 | Highly desirable  (P & R) | Conducting sensitivity analysis | Use sensitivity analyses to assess the robustness of results, such as the impact of notable assumptions, imputed data, borderline decisions, and studies at high risk of bias or with poor quality. | It is important to be aware when results are robust, since the strength of the conclusion may be strengthened or weakened. | 9.7 | Methods, page 14, subheading “Sensitivity analysis” |
| C72 | Mandatory  (R) | Interpreting results | Interpret a statistically non-significant p-value (e.g. larger than 0.05) as a finding of uncertainty unless confidence intervals are sufficiently narrow to rule out an important magnitude of effect. | Authors commonly mistake a lack of evidence of effect as evidence of a lack of effect. | 12.4.2  12.7.4 | Not applicable. This article is a review protocol. |
| C73 | Highly desirable  (R) | Investigating reporting biases | Consider the potential impact of reporting biases on the results of the review or the meta-analyses it contains. | There is overwhelming evidence of reporting biases of various types. These can be addressed at various points in the review. A thorough search, and attempts to obtain unpublished results, might minimize the risk. Analyses of the results of included studies, for example using funnel plots or regression tests for funnel plot asymmetry, can sometimes help determine the possible extent of the problem, as can attempts to identify study protocols, which should be a more routine feature of a review. | 10.1  10.2 | Not applicable. This article is a review protocol. |
| Summarizing the findings | | | | | |  |
| C74 | Optional  (P & R) | Including a ‘Summary of Findings’ table | Include a ‘Summary of Findings’ table according to recommendations described in Chapter 11 of the Cochrane Handbook (version 5 or later). Specifically:   include results for one population group  (with few exceptions);   indicate the intervention and the comparison intervention;   include seven or fewer patient-important outcomes;   describe the outcomes (e.g. scale, scores, follow-up);   indicate the number of participants and studies for each outcome;   present at least one baseline risk for each dichotomous outcome (e.g. study  population or median/medium risk) and  baseline scores for continuous outcomes (if appropriate);   summarize the intervention effect (if appropriate); and   include a measure of the quality of the body of evidence. | For co-registered reviews, a ‘Summary of Findings’ table is highly desirable. For those reviews, authors should justify why a ’Summary of Findings’ table is not included if this is the case. | 11.5 | Methods, page 14, subheading “Summary of findings and assessment of the certainty of the evidence”. |
| C75 | Optional  (P & R) | Use the GRADE approach to assess the body of evidence | If the review is co-registered with a Cochrane group, it is highly desirable to use the five GRADE considerations (study limitations, consistency of effect, imprecision, indirectness and publication bias) to assess the quality of the body of evidence for each outcome, and to draw conclusions about the quality of evidence within the text of the review. It is mandatory for all reviews to assess the quality of the body of evidence in some narrative or empirical manner; however, it is not mandatory that the GRADE approach be used to accomplish that goal. | GRADE is the most widely used system for summarizing confidence in effects of the interventions by outcome across studies. It is preferable to use the GRADE tool (as implemented in GRADEprofiler and described in the help system of the software). This should help to ensure that author teams are accessing the same information to inform their judgments. If the GRADE tool is used, the five GRADE considerations should be addressed irrespective of whether the review includes a ‘Summary of Findings’ table | 12.2 | Methods, page 14, subheading “Summary of findings and assessment of the certainty of the evidence”. |
| C76 | Optional  (R) | Justifying assessments of the quality of the body of evidence | Justify and document all assessments of the quality of the body of evidence (for example downgrading or upgrading if using the GRADE tool). | By adopting a structured approach, transparency is ensured in showing how interpretations have been formulated and the result is more informative to the reader. | 12.2.1 | Not applicable. This article is a review protocol. |
| Reaching conclusions | | | | | |  |
| C77 | Mandatory  (R) | Formulating implications for practice | Base conclusions only on findings from the synthesis (quantitative or narrative) of studies included in the review. | The conclusions of the review should convey the essence of the synthesis of included studies, without selective reporting of particular findings on the basis of the result, and without drawing on data that were not systematically compiled and evaluated as part of the review. | 12.7.4 | Not applicable. This article is a review protocol. |
| C78 | Highly desirable  (R) | Avoiding recommendations | Avoid providing recommendations for practice. | Campbell reviews should not attempt to tell people which interventions should or should not be used, since local considerations may be relevant. However, the implications of the findings should be discussed, and decision-making can be helped by laying out different scenarios. | 12.7.2 | Not applicable. This article is a review protocol. |
| C79 | Highly desirable  (R) | Formulating implications for research | Structure the implications for research to address the nature of evidence required, including population intervention comparison, outcome, and type of study. | Anyone wishing to conduct a study in the topic area of the review should be provided with a clear sense of what the remaining uncertainties are. A useful framework for considering implications for research is EPICOT (evidence, population, intervention, comparison, outcome and time stamp). | 12.7.3 | Not applicable. This article is a review protocol. |

*These Handbook section numbers are specific to Version 5.1. All other section numbers apply equally to the 2008 edition (and 2009 reprints) published by Wiley-Blackwell.
