# Supplementary material for: Protocol: Factors contributing to the discontinuation of breastfeeding upon women's return to work: A systematic review protocol
Source: Campbell Syst Rev. 2024 Sep 9;20(3):e1434. doi: 10.1002/cl2.1434 (PMC11382015; doi:10.1002/cl2.1434)
Supplement: Supplementary file 2 — Supporting information. [file CL2-20-e1434-s003.docx]

| Suggested timetable to apply the protocol | |
| --- | --- |
| Activity | Proposed weeks |
| Planning and team setup | 1 |
| Literature search | 2 |
| Duplicate removal | 3-4 |
| Screening titles and abstracts of identified studies for eligibility | 5-7 |
| Full-text screening | 8-10 |
| Data extraction from selected manuscripts | 11-13 |
| Risk-of-bias assessment | 14 |
| Quantitative and qualitative data synthesis | 15-18 |
| Systematic review redaction | 19-20 |
| Submission of manuscript | 21 |
