# Supplementary material for: Protocol: Factors contributing to the discontinuation of breastfeeding upon women's return to work: A systematic review protocol
Source: Campbell Syst Rev. 2024 Sep 9;20(3):e1434. doi: 10.1002/cl2.1434 (PMC11382015; doi:10.1002/cl2.1434)
Supplement: Supplementary file 3 — Supporting information. [file CL2-20-e1434-s002.docx]

I

| Table 1. Search strategy for a search conducted on August 8, 2024. | | |
| --- | --- | --- |
| Search engine or database | Query | Results |
| PubMed | (("breast feeding"[MeSH Terms] OR ("lactation"[MeSH Terms] OR "breast feeding"[MeSH Terms])) AND ("Return to Work"[MeSH Terms] OR "work return"[Title/Abstract:~3] OR "return to Employment"[All Fields] OR "return to job"[All Fields] OR "parental leave"[MeSH Terms])) AND (english[Filter] OR spanish[Filter])  *Filters* - English, Spanish | 398 |
| MEDLINE* | ((Breast Feeding or lactation) and ("Return to Work" or "return to employment" or "return to job" or ("Maternity Leave" or "paternal leave" or "working mother"))).mp. [mp=title, book title, abstract, original title, name of substance word, subject heading word, floating sub-heading word, keyword heading word, organism supplementary concept word, protocol supplementary concept word, rare disease supplementary concept word, unique identifier, synonyms, population supplementary concept word, anatomy supplementary concept word] | 559 |
| Academic Search Complete** | AB ((breast feeding OR lactation OR "breast-feeding" OR "lactation support") AND ((”Return to Work” OR return to employment OR return to job OR "job resumption" OR "employment reentry") OR ("Maternity Leave" OR "parental leave" OR "family leave" OR "childcare leave")))  *Amplifiers* - Apply related words  *Search mode* - Find all my search terms | 153 |
| Applied Science & Technology Full Text (H.W. Wilson)** | AB ((breast feeding OR lactation OR "breast-feeding" OR "lactation support") AND ((”Return to Work” OR return to employment OR return to job OR "job resumption" OR "employment reentry") OR ("Maternity Leave" OR "parental leave" OR "family leave" OR "childcare leave")))  *Amplifiers* - Apply related words  *Search mode* - Find all my search terms | 1 |
| Biological & Agricultural Index Plus (H.W. Wilson)** | AB ((breast feeding OR lactation OR "breast-feeding" OR "lactation support") AND ((”Return to Work” OR return to employment OR return to job OR "job resumption" OR "employment reentry") OR ("Maternity Leave" OR "parental leave" OR "family leave" OR "childcare leave")))  *Amplifiers* - Apply related words  *Search mode* - Find all my search terms | 1 |
| Business Source Premier** | AB ((breast feeding OR lactation OR "breast-feeding" OR "lactation support") AND ((”Return to Work” OR return to employment OR return to job OR "job resumption" OR "employment reentry") OR ("Maternity Leave" OR "parental leave" OR "family leave" OR "childcare leave")))  *Amplifiers* - Apply related words  *Search mode* - Find all my search terms | 32 |
| Dentistry & Oral Sciences Source** | AB ((breast feeding OR lactation OR "breast-feeding" OR "lactation support") AND ((”Return to Work” OR return to employment OR return to job OR "job resumption" OR "employment reentry") OR ("Maternity Leave" OR "parental leave" OR "family leave" OR "childcare leave")))  *Amplifiers* - Apply related words  *Search mode* - Find all my search terms | 2 |
| Fuente Académica Premier** | AB ((breast feeding OR lactation OR "breast-feeding" OR "lactation support") AND ((”Return to Work” OR return to employment OR return to job OR "job resumption" OR "employment reentry") OR ("Maternity Leave" OR "parental leave" OR "family leave" OR "childcare leave")))  *Amplifiers* - Apply related words  *Search mode* - Find all my search terms | 2 |
| MedicLatina** | AB ((breast feeding OR lactation OR "breast-feeding" OR "lactation support") AND ((”Return to Work” OR return to employment OR return to job OR "job resumption" OR "employment reentry") OR ("Maternity Leave" OR "parental leave" OR "family leave" OR "childcare leave")))  *Amplifiers* - Apply related words  *Search mode* - Find all my search terms | 4 |
| Open Dissertations** | AB ((breast feeding OR lactation OR "breast-feeding" OR "lactation support") AND ((”Return to Work” OR return to employment OR return to job OR "job resumption" OR "employment reentry") OR ("Maternity Leave" OR "parental leave" OR "family leave" OR "childcare leave")))  *Amplifiers* - Apply related words  *Search mode* - Find all my search terms | 9 |
| Latin American and Caribbean Health Sciences Literature (LILACS)^✝^ | (breastfe* OR "breast feeding" OR lactation) AND ((return TO work OR "return to work") OR (maternity leave OR parental leave OR working mothers)) AND ( la:("en" OR "es"))  *Search in* Title, abstract, subject  *Filters* - English, Spanish | 120 |
| Index Medicus for the Eastern Mediterranean Region (IMEMR)^✝^ | (breastfe* OR "breast feeding" OR lactation) AND ((return TO work OR "return to work") OR (maternity leave OR parental leave OR working mothers)) AND ( la:("en" OR "es"))  *Search in* Title, abstract, subject  *Filters* - English, Spanish | 31 |
| Index Medicus for South-East Asia Region (IMSEAR)^✝^ | (breastfe* OR "breast feeding" OR lactation) AND ((return TO work OR "return to work") OR (maternity leave OR parental leave OR working mothers)) AND ( la:("en" OR "es"))  *Search in* Title, abstract, subject  *Filters* - English, Spanish | 25 |
| Western Pacific Region Index Medicus (WPRIM)^✝^ | (breastfe* OR "breast feeding" OR lactation) AND ((return TO work OR "return to work") OR (maternity leave OR parental leave OR working mothers)) AND ( la:("en" OR "es"))  *Search in* Title, abstract, subject  *Filters* - English, Spanish | 17 |
| Abridged Index Medicus or “Core Clinical” (AIM)^✝^ | (breastfe* OR "breast feeding" OR lactation) AND ((return TO work OR "return to work") OR (maternity leave OR parental leave OR working mothers)) AND ( la:("en" OR "es"))  *Search in* Title, abstract, subject  *Filters* - English, Spanish | 9 |
| Epistemonikos | (title:((breastfe* OR "breast feeding" OR lactation) AND (("return to work" OR return to work OR return to job) OR ("maternity leave" OR "parental leave" OR "working mother"))) OR abstract:((breastfe* OR "breast feeding" OR lactation) AND (("return to work" OR return to work OR return to job) OR ("maternity leave" OR "parental leave" OR "working mother")))) | 112 |
| Cochrane Database of Systematic Reviews^‡^ | ("breast feeding" OR breastfe* OR lactation) AND ((return to work OR return to job) OR (Parental Leave OR maternity leave OR working mother))  *Search in* Title, abstract, keyword  *Amplifiers* - Word variations have been searched | 21 |
| [Cochrane Central Register of Controlled Trials](https://www.cochranelibrary.com/central/about-central) (CENTRAL)^‡^ | (("breast feeding" OR breastfe* OR lactation) AND ((return to work OR return to job) OR (Parental Leave OR maternity leave OR working mother))):ti,ab,kw  *Search in* Title, abstract, keyword  *Amplifiers* - Word variations have been searched | 465 |
| Campbell Systematic Reviews | ("breast feeding" OR breastfeeding OR "lactation") AND (("return to work" OR "return to employment" OR "return to job") OR ("maternity leave")) | 4 |
| International Clinical Trials Registry Platform (ICTRP) | (Breast Feeding OR lactation) and (Return to Work OR return to employment OR return to job OR Maternity Leave)  *Search with synonyms* | 1 |
| EU Clinical Trials Register | ("breast feeding" OR breastfeeding OR "lactation") AND (("return to work" OR "return to employment" OR "return to job") OR ("maternity leave")) | 0 |
| Scielo | (breastfe* OR "breast feeding" OR lactation) AND ((return TO work OR "return to work") OR (maternity leave OR parental leave OR working mothers)) | 248 |
| Open Access Theses and Dissertations | abstract:(breastfe* OR "breast feeding" OR lactation) AND (("return to work") OR (maternity leave OR parental leave)) | 768 |
| *Note:* *= accessed through Ovid; **= accessed through EBSCOhost; ^✝^= accessed through the Virtual Health Library; ^‡^= accessed through The Cochrane Library | | |
