# Supplementary material for: Protocol: Factors contributing to the discontinuation of breastfeeding upon women's return to work: A systematic review protocol
Source: Campbell Syst Rev. 2024 Sep 9;20(3):e1434. doi: 10.1002/cl2.1434 (PMC11382015; doi:10.1002/cl2.1434)
Supplement: Supplementary file 4 — Supporting information. [file CL2-20-e1434-s001.docx]

| **Full citation:** | |
| --- | --- |
| **Gender of authors:** | |
| **Year:** | |
| **Country:** | |
| **e-mail of the Corresponding author:** | |

| **Inclusion in review:⃞YES ⃞NO** | |
| --- | --- |
| ***Criteria for exclusion (choose all that apply):***   - Participants: Does not include cisgender women who breastfeed and return to work. - Concept: Does not study early breastfeeding cessation. - Context: Does not study return to paid work activities. - Type of source: Not a quantitative primary study. - Availability: Full study not available | |

| ***Source type***   - Primary study in scientific journal - Conference proceedings - Thesis - Dissertation - Other: _____________________ |
| --- |

| ***Methodology***  Study design:   - Descriptive - Correlational - Quasi-experimental - Experimental   Study type:   - Randomized controlled trial - Non-randomized controlled trial - Non-controlled trial - Cohort - Case-control   Study follow-up:   - Retrospective - Cross-sectional - Prospective |
| --- |

| ***Risk-of-bias assessment:***  For randomized controlled trials:   - Low risk of bias - Some concern of risk of bias - **High risk of bias**   For nonrandomized intervention studies:   - Low risk of bias - Moderate risk of bias - Serious risk of bias - **Critical risk of bias**   For cohort studies, case-control studies, and analytical cross-sectional studies:   - Low risk of bias - Unclear risk of bias - **High risk of bias** |
| --- |

| ***Participants:***   - Number of participants: ___________________ - Number of losses: ___________________ - Age: ___________________ - Nationality: ___________________ - Ethnicity: ___________________ - Education: ___________________ - Socioeconomic status: ___________________ - Parity: ___________________ |
| --- |

| ***Outcome:***   - Type of breastfeeding:   ⃞Exclusive (number of women):  ⃞Partial (number of women):   - Time since birth:   ⃞Mean:  ⃞Range (min-max): |
| --- |

| ***Context:***   - Return to work timing: ___________________ - Type of work / job position: ___________________ - Type of employment   ⃞Formal (number of women): ⃞Informal (number of women): ⃞N/A  ⃞Dependent (number of women): ⃞Self-employed (number of women): ⃞N/A  ⃞Full time (number of women): ⃞Part-time (number of women): ⃞N/A |
| --- |

***Extracted results:***

Note: Cases are defined as women who discontinued exclusive or partial breastfeeding in infants younger than six months, or any breastfeeding for older infants.

|  | **Number of women** | | | |
| --- | --- | --- | --- | --- |
|  | **Cases with risk factor** | **Cases without risk factor** | **Non-cases with risk factor** | **Non-cases without risk factor** |
| - Individual factors:   1.  2.  3.  4.  5.   - Interpersonal factors:   1.  2.  3.  4.  5.   - Community factors:   1.  2.  3.  4.  5.   - Institutional factors:   1.  2.  3.  4.  5.   - Public policies:   1.  2.  3.  4.  5. |  |  |  |  |

| ***Missing data:***  Contact the corresponding author for missing data request?   - Yes - No   Describe missing data to request: ___________________________________________________________________________________________________________________________________________________________________________________________________________________________________________________ |
| --- |
